# Folecitin Isolated from *Hypericum oblongifolium* Exerts Neuroprotection against Lipopolysaccharide-Induced Neuronal Synapse and Memory Dysfunction via p-AKT/Nrf-2/HO-1 Signalling Pathway

**DOI:** 10.1155/2022/9419918

**Published:** 2022-03-28

**Authors:** Umar Farooq, Muhammad Umar Khayam Sahibzada, Taous Khan, Rahim Ullah, Muhammad Shahid, Ameer Khusro, Veronique Seidel, Magda H. Abdellattif, Talha Bin Emran

**Affiliations:** ^1^Department of Pharmacy, COMSATS University Islamabad Abbottabad Campus, Abbottabad, Pakistan; ^2^Department of Pharmacy, Sarhad University of Science & Information Technology, Peshawar 25100, Khyber Pakhtunkhwa, Pakistan; ^3^Department of Pharmacy, University of Peshawar, Peshawar, Pakistan; ^4^Department of Pharmacy, Institute of Integrative Biosciences, CECOS University of IT and Emerging Sciences, Peshawar, Khyber Pakhtunkhwa, Pakistan; ^5^Research Department of Plant Biology and Biotechnology, Loyola College, Chennai, Tamil Nadu, India; ^6^Natural Products Research Laboratory, Strathclyde Institute of Pharmacy and Biomedical Sciences, University of Strathclyde, Glasgow G4 0RE, UK; ^7^Department of Chemistry, College of Science, Taif University, PO. Box 11099, Taif 21944, Saudi Arabia; ^8^Department of Pharmacy, BGC Trust University Bangladesh, Chittagong 4381, Bangladesh

## Abstract

Neurodegenerative diseases, especially Alzheimer's disease (AD), are characterised with neuronal synapse and memory dysfunction, and thus, there is an urgent need to find novel therapeutic medicines that can target different pathways to restore the deficits. In this investigation, we assessed the medicinal potency of folecitin (a flavonoid isolated from *Hypericum oblongifolium* Wall.) against lipopolysaccharide (LPS)-induced amyloidogenic amyloid beta (A*β*) production pathway-mediated memory impairment in mice. The LPS was administered intraperitonially (i.p.) 250 *μ*g/kg/day for 3 consecutive weeks, followed by the coadministration of folecitin (30 mg/kg/day) with LPS for the last two weeks (2^nd^ and 3^rd^ week). The expression of various proteins involved in synapse, neuronal death, and A*β* generation was evaluated using the Western blot approach. Results indicated that folecitin significantly decreased LPS-induced apoptotic proteins; expressed BAX, PARP-1, and caspase-3 proteins; and inhibited BACE1 that cleaves transmembrane amyloid precursor protein and the amyloidogenic A*β* production pathway. Folecitin restored both preneural and postneuronal synapse, accompanied by the improvement in memory impairment. Moreover, folecitin significantly activated endogenous antioxidant proteins Nrf-2 and HO-1 by stimulating the phosphorylation of Akt proteins. These findings indicate that folecitin might be a promising target for developing novel medication to treat neurodegenerative disorders caused by neurotoxins.

## 1. Introduction

Neurological disorders (NDs), for example, Parkinson's disease (PD), Alzheimer's disease (AD), and amyotrophic lateral sclerosis (ALS), are the key progressive neurodegenerative problems worldwide. According to reports, 46.8 million individuals suffered from NDs in 2015, incurring an estimated treatment cost of US$ 818 billion. By 2040, NDs are expected to be the 2^nd^ most common cause of death [1]. The NDs are characterised by the depletion (or inadequate synthesis) of neurotransmitters, inflammation, aggregation of misfolded proteins, and oxidative stresses in central nervous system (CNS), for instance, *β*-amyloid and Tau proteins in AD [2, 3]. The treatment of AD uses various therapeutic agents of natural and synthetic origin [4], some of which such as the acetylcholinesterase inhibitors present adverse side effects [5]. Other treatments such as anti-Tau protein and *β*-amyloid antibodies, and *β*-secretase 1 (BACE1) inhibitors are very expensive [6, 7].

Plants have a long history of contributing to the discovery of new drugs. There are approximately 300,000 different species of higher plants worldwide, of which more than 85% have not been explored for the presence of bioactive principles [8, 9]. Plants produce a diverse range of chemicals (e.g., alkaloids, polyphenols, flavonoids, glycosides, terpenoids, and saponins) that exhibit various biological properties [10, 11]. In Asian countries, more than 120 traditional plants-based medicines are used for the management of CNS disorders [12–14]. This includes extracts and phytoconstituents from various plants such as *Panax ginseng* (ginsenosides) [15], *Curcuma longa* (curcumin) [16], *Hypericum perforatum* (hyperoside) [17], *Centella asiatica* (catechin) [18], *Bacopa monneri* (bacosides) [19], *Withania somnifera* (withanolides) [20], and *Ginkgo biloba* (ginkgolides) [21] that exhibited promising psychotropic and neuroprotective properties.

The genus *Hypericum* (Hypericaceae) consists of herbs and shrubs found commonly in temperate regions and used as a source of natural medicines, pigments, dyes, gums, resins, and timbers [22]. Various species within the *Hypericum* genus, especially *Hypericum caprifoliatum* [23], *Hypericum perforatum* [24, 25], *Hypericum grandifolium* [26], *Hypericum oblongifolium* [27], *Hypericum polyanthemum* [28], and *Hypericum triquetrifolium* [29] have been studied for their antidepressant, antioxidant, anxiolytic, antimicrobial, antiviral, anticancer, anti-inflammatory, and antiulcerogenic properties.

*H. oblongifolium* Wall. (also known as basant, sheen chayi, and Pendant St. John's Wort) grows at altitudes of 800–1200 m within the Western Himalayas [27]. It is widely distributed in Northern Pakistan. Its flowers are yellow with persistent-withering petals, and they bloom from June to September [30]. The species is used traditionally for external wounds, hepatitis, gastric ulcers, and other gastrointestinal disorders [31]. It has potent *in vitro* antiglycation and antioxidant properties [32]. It has also demonstrated antiproliferative activity on HT-29 human colon adenocarcinoma cells [33]. The species mainly contains flavonoids, triterpenes, and xanthones [34].

Flavonoids are a vast category of natural polyphenolic plant pigments found in a wide variety of foods, including fruits, cereals, herbs, and drinks. Flavonoids have anticancer, cardiovascular, antioxidant, neuroprotective, and anti-inflammatory effects [35, 36]. Flavonoids are considered as potential neuroprotective compounds that can modulate cellular mechanisms implicated with neurodegeneration [35, 37–39]. Flavonoids show the characteristics of both antioxidant and signal pathway modulator. It can modulate cellular signal cascades by interacting with enzymes or receptors that are involved in activation and deactivation of signalling pathways [40, 41]. Reports suggest that a habitual intake of dietary flavonoids can reduce the risk of dementia and stroke [39]. For instance, flavonoids in fruits, vegetables, grains, etc. seem to prevent or reverse cognitive related deficits [37]. Considering the pivotal role of flavonoids, in this study, we focused on the isolation of folecitin (a flavonoid), from *H. oblongifolium*, and the evaluation of its protective activity against neuroinflammation using a lipopolysaccharide (LPS)-induced neurotoxicity assay in mice.

## 2. Materials and Methods

### 2.1. Plant Materials

Fresh leaves of *H. oblongifolium* were collected from Thandiyani, Abbottabad, KPK, Pakistan. The plant was authenticated by Dr. Banaras Khan, Department of Botany, Post Graduate College Attock city, and a voucher specimen (Atk/102/2018) was deposited in the herbarium of College.

### 2.2. Extraction

The pulverised leaves (15 kg) were macerated for 14 days in methanol:water solution (7 : 3) with frequent stirring using a steel rod. The resulting suspension was filtered by means of Whatman filter paper. The obtained solution was then concentrated under vacuum to afford a first crude extract. The remaining plant material was re-extracted with a fresh mixture of methanol:water (7 : 3) for another 7 days. After filtration, as aforementioned, the sample was concentrated under vacuum to afford a second extract. The combined crude extracts were resuspended in distilled water (1 L), and liquid-liquid partition was carried out using analytical grade solvents of increasing polarity starting with *n-*hexane, chloroform, ethyl acetate, and *n-*butanol to obtain *n-*hexane (640 g), chloroform (40 g), ethyl acetate (400 g), *n-*butanol (180 g), and aqueous (390 g) fractions.

### 2.3. Isolation of Folecitin from *H. oblongifolium*

Ethyl acetate fraction (400 g) was subjected to column chromatography over silica gel mesh size 230 (Merck), eluting with *n*-hexane:ethyl acetate having a ratio of 2 : 8. A total of 16 major fractions were obtained and pooled on the basis of their similar chemical profiles on thin layer chromatography (TLC) using silica gel 60 PF_254_ plates (Merck). Fraction 14 (1.2 g) was further subjected to flash silica gel column chromatography, eluting with ethyl acetate:chloroform (1 : 1). Twelve major fractions were pooled based on TLC analysis. Fractions 5–9 led to the separation of folecitin (70 mg) as yellow crystals. Folecitin was observed on TLC plates using cerium sulfate (CeSO_4_) and solid iodine, followed by heating. The structure of folecitin was determined following analysis of its ^1^H and ^13^C NMR, COSY, HSQC, and HMBC spectra recorded on Bruker spectrometers (Avance Av 500, 600/150 MHz) and comparison with the literature data [27]. All chemical shifts (*δ*) and coupling constants (J) were estimated in ppm and Hz.

### 2.4. Neuroprotective Activity

#### 2.4.1. Ethical Approval

All experimental animals were taken care of according to the approvals of the ethical committee of the Neuroprotective Medicine and Molecular Research Center, Ring Road Peshawar, Pakistan (Ethical committee number: NMMRC/2019/Rodents/015). Animals were handled as per the scientific procedures of the UK Animals guidelines Act 1986.

#### 2.4.2. Chemicals and Reagents

Lipopolysaccharide (LPS), phosphate buffered saline tablets, polyvinylidene fluoride (PVDF) membrane, reagent for tissue protein extraction (T-PER), RNAwait solution, reagent for protein assay dye, acrylamide, bis-acrylamide, sample buffer (2X Laemmli), ammonium per sulfate (APS), trizma base, TEMED, sodium dodecyl sulfate (SDS), glycine, methanol, KCl, skim milk, NaCl, and Tween 20 were purchased from Sigma Aldrich (USA) and stored at the required temperature for further experimental purposes.

#### 2.4.3. Maintenance of Animals Used

BALB/c adult male mice of 7-8 weeks old (weighing 30–32 g) were acquired from the Veterinary Research Institute, Peshawar, KPK, Pakistan. They were maintained separately in cages (Biobase, China), kept in a special room with a constant 12 h dark and 12 h light cycle at 27 ± 3°C, with easy access to food and water.

#### 2.4.4. Experimental Groups

Animals were arbitrarily divided into 4 experimental groups (*n* = 6). The total span of the experiment was 3 weeks (day 1 to day 21). The control group (C) received intraperitoneally (i.p.) normal saline for 3 consecutive weeks. Group 2 (LPS group) received LPS (i.p.) (250 *μ*g/kg) for 21 consecutive days. Group 3 (LPS + folecitin) received LPS (250 *μ*g/kg/day) for first 7 days and then continue till to the last day, while LPS was complemented by folecitin (30 mg/kg) from day 8 to day 21 of the experiment. The fourth and final group received folecitin (30 mg/kg) alone for the last two weeks of the experiment.

#### 2.4.5. Behavioural Tests

Two well-known behavioural tests, the Y-maze and the Morris water maze (MWM), were conducted in order to find out the neuroprotective effect of folecitin on learning and remembrance behaviours in adult mice (*n* = 6).

*(1) Y-Maze Test*. The Y-Maze test consisted of 3 arms of 60 cm length, 12 cm diameter (width), and 22 cm height at an angle of 120° from each other [42, 43]. Briefly, all the experimental mice were allowed to receive trainings for the first 3 days (3 training trials per day). In each trial, the animals were trained for 10 min so that they could adapt to their new surroundings. After resting for 2 days, all the mice were permitted to search and walk around the Y-Maze for 8 min by keeping them in the center of the arms. A build-in camera with software was used to record the movements of mice, total arm entrances, and consecutive number of triplets. The percentage (%) of repetitions was estimated as per the equation below,

Repetition (%) = (consecutive sets of triplets/total number of arm entries − 2) × 100.

The changes and repetition (%) were associated positively with spatial functioning memory.

*(2) MWM Test*. The MWM test was implemented to determine the four-dimensional learning (spatial learning) and commemoration in adult mice [42]. The MWM device was made of a circular water tank of about 1.8 m in diameter and 0.6 m in height filled with normal tap water (25–28°C). Skimmed milk powder (1 kg) was added to make the water appearance opaque. A grey escape plastic platform (35.5 cm in height) was located 1 cm underneath the water surface nearby middle of any one of the four corners of the maze apparatus. This unique stage can be lowered to the bottom of the tank or raised to its typical position on the maze apparatus during behavioural learning and testing. A second stand (36.5 cm in height), black in colour, was raised 2 cm over the water layer during the first 3 days of training. The MWM was encircled by white curtains with fixed patterns in order to provide a configuration of three-dimensional signals. Observations were made using a video tracing system (HVS Image Analyzing VP-112) with the in-built software.

Briefly, all the experimental mice were allowed to receive 3 training trials (60 s per trial) per day for 3 successive days [43]. On each training test, an animal was released freely in the MWM from one of four equally spread-out starting positions round the border of the water tank. If any animal did not locate the escape stage within 80 s on any training session, it was guided and placed for 30 s on the platform. After resting for 2 days, all the animals were exposed to the latency to escape from the water maze (ﬁnding the submerged escape platform) was determined for each test day (four successive days). Again, after three-day rest, ﬁnal escape latency and probe tests were carried out to estimate memory association. The time consumed in the particular quadrant by every animal was recorded.

### 2.5. Western Blot

Western blotting was carried out according to the method reported by Badshah et al. [44] with minor alterations. The mice brains were collected as quickly as possible and then homogenized in a T-PER solution. The levels of proteins in all 4 groups were measured using a BioRad protein assay. For each of the 4 groups, 30 *μ*g of the protein content was run on a 15–20% SDS-PAGE. After completing the electrophoresis process, all the proteins were shifted to a PVDF membrane (Santa Cruz Biotech, USA) over transblot (Bio-Rad). Different primary monoclonal antibodies (Santa Cruz, CA, USA) such as caspase-3, Bcl-2-associated *X* protein (BAX), beta-secretase-1 (BACE-1), poly (ADP-ribose) polymerase-1 (PARP-1), amyloid beta (A*β*), synaptophysin (SYP), postsynaptic density protein 95 (PSD-95), and beta actin as well as HRP-conjugated secondary antibodies (Santa Cruz, CA, USA) were smeared. All results were visualised in a dark room on X-ray films.

### 2.6. Statistical Analysis

Results obtained were calculated as group mean ± SEM (standard error mean) and analysed using one-way or two-way analysis of variance (ANOVA) followed by suitable *post hoc* tests. All the statistical investigations were conducted using the GraphPad Prism-5 software. *P* values less than 0.05 were defined as significant.

## 3. Results

### 3.1. Effect of Folecitin on LPS-Induced Neuroapoptotic Protein Markers

**Figure 1:**
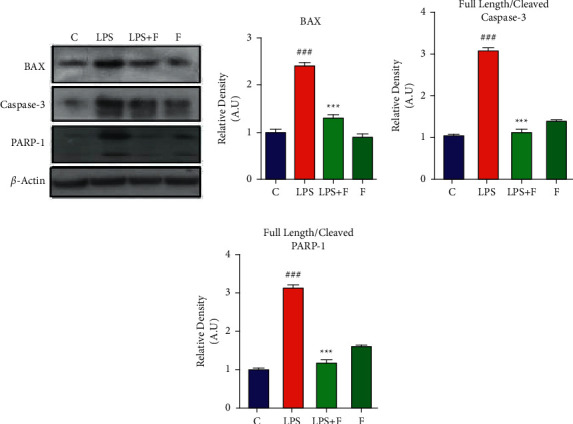
Folecitin decreased the expression of apoptotic protein markers in the mice brain. (a) Immunoblots of BAX, PARP-1, and caspase-3 in the brain homogenates of the adult mice for the experimental groups including (c) LPS, LPS + F, and F alone. Histogram of (b) BAX, (c) full-length/cleaved caspase-3, and (d) full-length/cleaved PARP-1. *β*-Actin was used as a loading control (reference control). The bands were analysed using the Image J software, and the density histograms (expressed in AU) compared to the control were organized using GraphPad Prism. Values are presented as the mean ± SEM for the indicated proteins (*n* = 6 mice per group). C: control, LPS: lipopolysaccharide, F: folecitin, AU: arbitrary units, ^###^*P* < 0.001 as compared to the C group, and ^*∗∗*^*P* < 0.01 and ^*∗∗∗*^*P* < 0.001^*∗∗∗*^*P* < 0.001 as compared to the LPS group.

Different apoptotic indicators such as BAX, caspase-3, and PARP-1 protein expressions were analysed with the Western blotting technique. Results revealed that a 3-week administration of LPS-induced widespread upregulation in the adult mice brain. The LPS induced the proapoptotic BAX protein expression (*P* < 0.01) and also triggered caspase-3 protein expression (*P* < 0.001). Finally, LPS-induced neuronal DNA fragmentation increases PARP-1 protein expression (*P* < 0.001). Interestingly, the administration of folecitin during the last 2 weeks of the experiment significantly inhibited proapoptotic-BAX (*P* < 0.001), followed by low levels of caspase-3 (*P* < 0.01) and PARP-1 (*P* < 0.001) proteins (Figure 1).

### 3.2. Effect of Folecitin on LPS-Induced A*β* Productions

**Figure 2:**
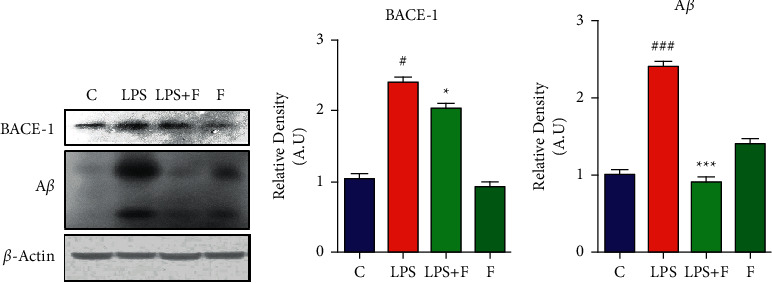
Folecitin reduced A*β* via inhibiting BACE-1 expression. (a) Immunoblots of BACE1 and A*β* in the brain homogenates of the adult mice for the experimental groups including (c) LPS, LPS + F, and F alone. Histogram of (b) BACE1 and (c) A*β*. *β*-Actin was used as a house-keeping loading control (reference control). The bands were analysed using the Image J software, and the density histograms (expressed in AU) compared to the control were organized using GraphPad Prism. Values are presented as the mean ± SEM for the indicated proteins (*n* = 6 mice per group). C: control, LPS: lipopolysaccharide, F: folecitin, AU: arbitrary units, ^#^*P* < 0.05 and ^###^*P* < 0.001 as compared to the C group, and ^*∗*^*P* < 0.05 and ^*∗∗∗*^*P* < 0.001 as compared to the LPS group.

The 3-week administration of LPS significantly increased beta-secretase activity with significant (*P* < 0.001) upregulation of the BACE-1 protein expression (which produces fragments of A*β* precursor protein to cut into toxic A*β* fragments). Similarly, the expression of A*β* protein (both oligomers and monomers) was significantly (*P* < 0.001) increased after LPS administration. In contrast, treatment with folecitin not only significantly (*P* < 0.05) inhibited BACE-1 expression but also significantly (*P* < 0.001) decreased both oligomers and monomers in the brain homogenates mixture of adult mice (Figure 2).

### 3.3. Effect of Folecitin on Neuronal Synapse

**Figure 3:**
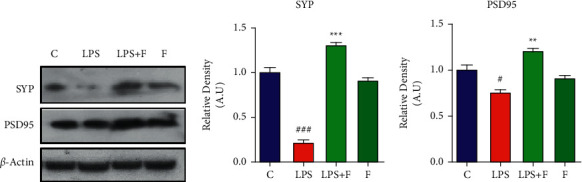
Folecitin reversed synaptic deficits induced by LPS. (a) Immunoblots of SYP and PSD95 proteins in the brain homogenates of male adult albino mice for the experimental groups including (c) LPS, LPS + F, and F alone. Histograms of (b) SYP and (c) PSD-95. *β*-Actin was used as a loading control (reference control). The bands were analysed using the Image J software, and the density histograms (expressed in AU) compared to the control were organized using GraphPad Prism. Values are presented as the mean ± SEM for the indicated proteins (*n* = 6 mice per group). C: control, LPS: lipopolysaccharide, F: folecitin, AU: arbitrary units, ^#^*P* < 0.05 and ^###^*P* < 0.001 ^.^as compared to the C group, and ^*∗∗*^*P* < 0.01 and ^*∗∗∗*^*P* < 0.001 as compared to the LPS group.

Western blot analysis revealed that systemic LPS injection reduced both SYP proteins (*P* < 0.001) and PSD-95 (*P* < 0.05) in the brain homogenates of mice with respect to the control group. Folecitin administration reduced the impact of LPS induction on synaptic markers and enhanced PSD-95 expression (*P* < 0.01), as compared to the LPS-treated group, and SYP (*P* < 0.001) (Figure 3).

### 3.4. Effect of Folecitin on Stimulation of Phosphorylated-Akt (p-Akt) to Activate Nrf-2/HO-1

**Figure 4:**
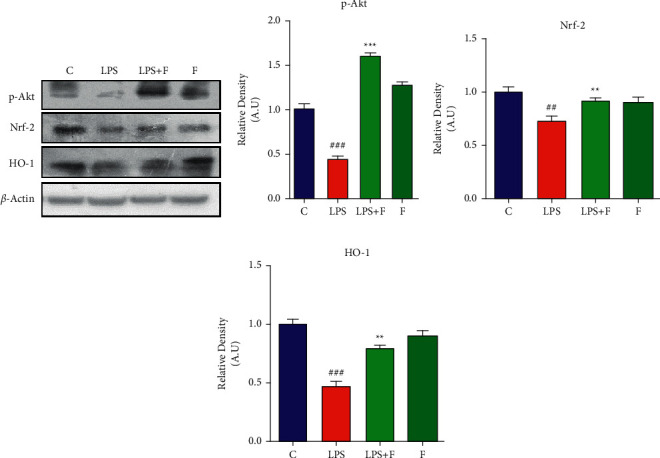
Folecitin stimulated the phosphorylated-Akt (p-Akt) to activate Nrf-2/HO-1. (a) Immunoblots of p-Akt, HO-1, and Nrf-2 proteins for the experimental groups including (c) LPS, LPS + F, and F alone. Histogram of (b) p-Akt, (c) Nrf-2, and (d) HO-1 proteins. *β*-Actin was used as a loading control (reference control). The bands were analysed using the Image J software, and the density histograms (expressed in AU) compared to the control were organized using GraphPad Prism. Values are presented as the mean ± SEM for the indicated proteins (*n* = 6 mice per group). C: control, LPS: lipopolysaccharide, F: folecitin, AU: arbitrary units, ^##^*P* < 0.01 and ^###^*P* < 0.001 as compared to the C group, and ^*∗∗*^*P* < 0.01 and ^*∗∗∗*^*P* < 0.001 as compared to the LPS group.

The LPS administration significantly suppressed the protein expressions of p-AKT (*P* < 0.001), nuclear factor erythroid 2-related factor 2 (Nrf-2) (*P* < 0.01), and HO-1 (*P* < 0.001), in comparison to the control group. In contrast, the administration of folecitin for two weeks significantly (*P* < 0.001) stimulated phospho-Akt protein. It was accompanied by the stimulation of Nrf-2 (*P* < 0.001) and HO-1 (*P* < 0.01) protein expression in the brain homogenates of mice (Figure 4).

### 3.5. Effect of Folecitin on Behaviour and LPS-Induced Memory Deficits

**Figure 5:**
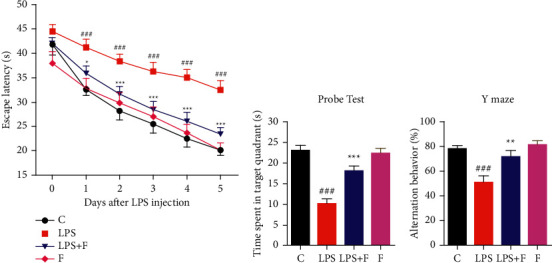
Improvement of LPS-induced memory impairment by folecitin. (a) Effect of folecitin on escape latency in the MWM test. (b) Effect of folecitin in the probe test (time consumed in the target quadrant on final day of the experiment) showed during the MWM test when the immersed platform was removed. (c) Effect of folecitin on alternation behaviour in the Y-maze test. Values are presented as the mean ± SEM (*n* = 6 mice per group). C: control, LPS: lipopolysaccharide, F: folecitin, ^###^*P* < 0.001 as compared to the control group, and ^*∗*^*P* < 0.05, ^*∗∗*^*P* < 0.01, and ^*∗∗∗*^*P* < 0.001 as compared to the LPS group.

The MWM and Y-maze tests were employed to check the memory-improving ability of folecitin. In the MWM test, LPS-treated mice showed significantly (*P* < 0.001) higher mean escape latencies from day 1 to day 5. There was a gradual decrease in the mean escape latency from the start to the end of the experiment, suggesting that these mice had impaired memory. There was a little decrease in the mean latency on a daily basis from day 1 to day 5. On the other hand, the mice treated with LPS + folecitin showed better memory from day 1 to day 5. The performance of these mice was better with reduced mean escape latencies from day 1 to day 5 compared to LPS-treated mice (*P* < 0.05, *P* < 0.001). The experimental mice treated with folecitin alone showed escape latencies similar to the control group from the beginning to the end of the test, suggesting that they have no memory deficiency (Figure 5(a)). In the probe test, the control animal spent more time as compared to the LPS-treated animals. Among the other 2 groups, the mice treated with LPS + folecitin had spent more time (*P* < 0.001) in the target quadrant, while the only folecitin-treated mice shown good memory by spending more time in the target quadrant (Figure 5(b)). The short-term memory was investigated by performing the Y-maze test. The control animals showed higher percentage of spontaneous alteration, while the LPS-treated animals displayed less percentage of spontaneous alteration (*P* < 0.001). Similarly, animals treated with LPS + folecitin showed a significantly (*P* < 0.01) higher percentage of spontaneous alteration, similar to the LPS-treated mice (Figure 5(c)).

## 4. Discussion

The current study reported that folecitin abrogated LPS-induced neuroapoptosis-mediated neuronal synapse dysfunction and memory impairment in the adult albino mice brain. Moreover, folecitin stimulated the p-Akt signalling pathway to activate Nrf-2 and its downstream molecule HO-1 in the brain of adult mice.

Alzheimer's disease is a neurodegenerative disorder with leading reason of death and disability [45]. In aging individuals, AD leads to a gradual impairment in cognition and neuronal dysregulation of communication. There are many evidences of involvement of neuroinflammation, apoptosis, and oxidative stress in the pathogenesis of AD and its associated neurodegenerative diseases [46]. In particular, it has been stated that the microglial activation plays a key role in triggering extreme oxidative stress when high levels of reactive oxygen species (ROS) are present [47]. Intracellular ROS in microglial cells causes inflammation and ultimately leads to neuronal cell death [48]. The level of endogenous ROS can be controlled by antioxidant molecules and antioxidative enzymes *via* the Nrf-2/antioxidant response element signal pathways [49].

Many reports conferred that the inclusion of LPS in rodents caused impairment of cognitive function and enhanced the level of AD-like markers, namely, A*β* and BACE-1 [44]. Several studies had reported that LPS impaired learning and memory [50, 51]. In the line with those studies, in the current report, the administration of LPS also impaired spatial learning and memory in the MWM test [44]. The MWM test results indicated that the mice receiving LPS had more mean escape time latencies and travelled longer distance to identify the escape platform. Our probe trial findings too demonstrated that LPS receiving animals' group failed in finding the exact location of the submerged platform and which is why they spent less time in the target quadrant. Animals receiving folecitin displayed a good recognition pattern and recognized the platform place and, hence, spent more time in the target quadrant.

The present study also demonstrated that folecitin significantly reduced apoptotic markers (BAX, caspase-3, and PARP-1 proteins) against LPS in the mice brain. Moreover, we hypothesized that folecitin had activated Nrf-2/HO-1 against LPS-induced oxidative stress in the brain of adult mice. Our results indicated that folecitin increased the nuclear translocation of Nrf-2/HO-1 and its production to inhibit LPS-induced oxidative stress. Of different genes involved in the antioxidative characteristics, HO-1 has been rated high, as it can exert protection via various mechanisms.

Akt acts as a signalling pathway with various functions, such as apoptosis, cell proliferation, and cellular defence, and is known to modulate Nrf-2 [52]. Cross-talk between the PI3K/Akt and Nrf-2 signalling pathways is capable of protecting cells against inflammatory and oxidative damage [53]. Subsequently, we investigated whether the Akt pathway-mediated folecitin-induced Nrf-2 activation and HO-1 expression or not. The results showed that folecitin induced a significant augmentation of the Akt phosphorylation pathway, which is directly associated with Nrf-2-mediated antioxidant response.

**Figure 6:**
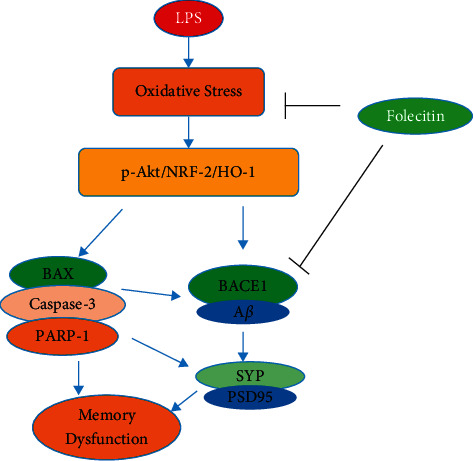
Proposed mechanism of folecitin neuroprotection action against LPS-induced oxidative stress mediated memory impairment. Folecitin activated the p-Akt/HO-1/Nrf-2 signalling pathway, leading to a reduction in oxidative stress, neurodegeneration, and associated neuronal synapse and memory impairment.

In this context, we used folecitin in the animal model of LPS to know its capability to reverse memory impairment. Our results revealed that folecitin significantly enhanced the LPS-inhibited endogenous antioxidant system, i.e., Nrf-2 and HO-1 accompanied by subsequent reduced expression of apoptotic markers in adult albino mice. It is followed by the positive effect on neuronal synapse and improvement in the memory impairment in the adult animal model. Most importantly, folecitin restored the p-Akt/Nrf-2/HO-1 signalling pathway to overcome the LPS-induced amyloidogenic pathway of A*β* production in male adult mice. Considering the antioxidative potential and vital ability in enhancing memory deficits, folecitin could be the potential drug candidates against neurodegenerative disorders, especially AD. An overview of the proposed mechanisms of action of folecitin is illustrated in Figure 6.

## 5. Conclusions

The administration of LPS to the brain of adult mice caused neuronal synapse and memory dysfunction. On the other hand, folecitin significantly abrogated the LPS-induced amyloidogenic A*β* production and improved the associated neuronal synapse and memory impairment. Moreover, folecitin restored the p-Akt signalling pathway to activate Nrf-2/HO-1 signalling. Owing to its neuroprotective potential and ability to improve memory deficits, folecitin might be a promising lead in designing new drugs for neurotoxin-triggered neurodegenerative problems [54].

## Data Availability

All the data are included within this manuscript.
